# *EPHA6* rs4857055 C > T polymorphism associates with hypertension through triglyceride and LDL particle size in the Korean population

**DOI:** 10.1186/s12944-017-0620-5

**Published:** 2017-12-06

**Authors:** Minjoo Kim, Hye Jin Yoo, Minkyung Kim, Jiyoo Kim, Seung Han Baek, Min Song, Jong Ho Lee

**Affiliations:** 1 Research Center for Silver Science, Institute of Symbiotic Life-TECH, Yonsei University, Seoul, 03722 Korea; 2 Department of Food and Nutrition, Brain Korea 21 PLUS Project, College of Human Ecology, Yonsei University, Seoul, 03722 Korea; 3 National Leading Research Laboratory of Clinical Nutrigenetics/Nutrigenomics, Department of Food and Nutrition, College of Human Ecology, Yonsei University, Seoul, 03722 Korea; 4 Institute of Convergence Technology, Yonsei University, Seoul, 03722 Korea; 5 Department of Library and Information Science, Yonsei University, Seoul, 03722 Korea

**Keywords:** *EPHA6*, Polymorphism, Hypertension, Triglyceride, LDL particle size

## Abstract

**Background:**

Erythropoietin-producing human hepatocellular (Eph) receptors might contribute to the development of atherosclerosis. A genome-wide association study indicated that the Eph receptor A6 gene (*EPHA6*) associated with at least 1 blood pressure (BP) phenotype. The objective of the present study was to determine whether *EPHA6* is a novel candidate gene for hypertension in a Korean population.

**Methods:**

A total 2146 study participants with normotension and hypertension were included. Genotype data were obtained using a Korean Chip. To assess the association between single-nucleotide polymorphisms (SNPs) and BP, we performed a linear regression analysis, which showed that rs4850755 in the *EPHA6* gene was the SNP most highly associated with both systolic and diastolic BP.

**Results:**

The presence of the TT genotype of the *EPHA6* rs4857055 C > T SNP was associated with a higher risk of hypertension after adjusting for age, sex, body mass index (BMI), smoking, and drinking [odds ratio 1.533, *P =* 0.001]. In the control group, significant associations were observed between systolic BP and the rs4857055 polymorphism and between diastolic BP and the rs4857055 polymorphism. In the hypertension group, a significant association was observed between systolic BP and the rs4857055 polymorphism. In the hypertension group, subjects with the TT genotype showed significantly higher systolic BP than CC subjects. Additionally, in the hypertension group, TT carriers showed a higher tendency of serum triglyceride (*P* = 0.069) and significantly higher apolipoprotein B (*P* = 0.015) and smaller low-density lipoprotein (LDL) particle size (*P* < 0.001) than either TC or CC subjects.

**Conclusions:**

These results could suggest that the *EPHA6* rs4857055 C > T SNP is a novel candidate gene for hypertension in the Korean population. Additionally, the TT genotype could be associated with hypertriglyceridemia and small LDL particle size in hypertension.

**Electronic supplementary material:**

The online version of this article (10.1186/s12944-017-0620-5) contains supplementary material, which is available to authorized users.

## Background

Hypertension is one of the well-known risk factors for atherosclerotic vascular diseases. As a complex trait, hypertension develops due to both genetic and environmental factors [1]. In fact, evidence from family studies has indicated that more than 30% of blood pressure (BP) variations could be attributed to genetics [2, 3]. Recently, genome-wide association studies (GWAS) identified more than 50 single-nucleotide polymorphisms (SNPs) associated with an increased risk of hypertension [4–6].

Erythropoietin-producing human hepatocellular (Eph) receptors are a group of receptors that are activated in response to binding with Eph receptor-interacting proteins (ephrins) [7, 8]. Ephs have important roles in a variety of biological functions. For instance, Sakamoto et al. [9] indicated that a wide variety of ephrins and Eph receptors might affect monocyte chemotaxis, contributing to the development of atherosclerosis. As such, the Eph/ephrin molecule is important for the developing cardiovascular system, as shown by the presence of heart and blood vessel defects resulting from knockout of Eph receptors or ephrins [10]. Eph receptors/ephrins expressed in blood vessels and their counterparts in immune cells are associated with inflammatory processes ranging from increased endothelial permeability and the mediation of inflammatory cell adhesion and migration to atherosclerotic plaque development [11–14]. Furthermore, EphB6, ephrin-B1, and ephrin-B3 expressed in vascular smooth muscle cells (VSMCs) can contribute to BP regulation [15, 16], and an Eph receptor B6 gene (*Ephb6*) knockout mouse model exhibited higher BPs than their wild-type counterparts [17]. A genome-wide gene-based analysis identified the Eph receptor A6 gene (*EPHA6*) as associated with at least 1 BP phenotype in a recent study of genome-wide gene-sodium interaction analyses on BP [18]. Because a close relationship exists between Ephs/ephrins and BP, particularly between *EPHA6* and BP [18], specific *EPHA6* SNP genotypes in humans could be associated with BP. The Korean Chip (K-CHIP) was developed as a low-cost customized chip that is optimized for genetic studies on disease and complex traits in the Korean population. Therefore, the objective of the present study was to determine whether *EPHA6* is a novel candidate gene for hypertension in the Korean population.

## Methods

### Study population

A total of 2167 study participants with nondiabetic normotension (systolic BP < 140 mmHg and diastolic BP < 90 mmHg) and hypertension (systolic BP ≥ 140 mmHg or diastolic BP ≥ 90 mmHg) aged 20–86 years were recruited from the Health Service Center during routine checkups at the National Health Insurance Corporation Ilsan Hospital, Goyang, Korea (January 2010–March 2015), for this study. Based on the data screened from the Health Service Center, subjects who agreed to participate in the study were referred to the Department of Family Medicine. The health of potential subjects was reassessed, and subjects who met the study criteria were included. The exclusion criteria were a current diagnosis and/or history of cardiovascular disease, liver disease, renal disease, pancreatitis, or cancer; pregnancy or lactation; and regular use of any medication. The aim of the study was carefully explained to all of the participants, who provided their written informed consent. The Institutional Review Board of Yonsei University and the National Health Insurance Corporation Ilsan Hospital approved the study protocol, which complied with the Declaration of Helsinki.

### Clinical and biochemical assessments

Detailed information on the clinical and biochemical assessments is provided elsewhere [19]. The subjects’ body weights, heights, and waist circumferences were measured, and their body mass indexes (BMIs) were calculated in units of kilograms per square meter (kg/m^2^). BP was measured using a random-zero sphygmomanometer (HM-1101, Hico Medical Co., Ltd., Chiba, Japan) with appropriately sized cuffs after a rest period of at least 20 min in a seated position. BP was measured three times in both arms. The differences between the three BP measurements were always <2 mmHg, and the average values for the systolic BP and diastolic BP measurements were used. Participants were instructed not to smoke or drink alcohol for at least 30 min before each BP measurement.

Blood samples were collected following an overnight fast of at least 12 h. The levels of fasting triglycerides, total cholesterol, high-density lipoprotein (HDL) cholesterol, low-density lipoprotein (LDL) cholesterol, glucose, insulin, and LDL particle size were measured as previously described [19]. Insulin resistance (IR) was determined with the homeostasis model assessment (HOMA) using the following equation: HOMA-IR = [fasting insulin (μIU/mL) × fasting glucose (mmol/L)] / 22.5. Serum apolipoprotein (apo) A-I and apo B levels were determined by turbidity at 340 nm using specific anti-serum (Roche, Basel, Switzerland).

### Affymetrix axiom™ KORV1.0–96 Array hybridization and SNP selection

A total of 2167 samples were genotyped according to the manufacturer’s protocol, which recommended the Axiom® 2.0 Reagent Kit (Affymetrix Axiom® 2.0 Assay User Guide; Affymetrix, Santa Clara, CA, USA). Approximately 200 ng of genomic DNA (gDNA) was amplified and randomly fragmented into 25- to 125-base pair (bp) fragments. The initial gDNA amplification was performed in a 40-μL reaction volume containing 20 μL of genomic DNA at a concentration of 10 ng/μL and 20 μL of a denaturation master mix. The initial amplification reaction was conducted as follows: 10 min at room temperature for the initial amplification; then, the incubated products were amplified with 130 μL of Axiom 2.0 Neutral Soln, 225 μL of Axiom 2.0 Amp Soln and 5 μL of Axiom 2.0 Amp Enzyme. The amplification reactions were performed for 23 ± 1 h at 37 °C. The amplification products were analyzed in an optimized reaction to amplify fragments between 200 and 1100 bp in length. A fragmentation step reduced the amplified products to segments approximately 25–50 bp in length, which were end-labeled using biotinylated nucleotides. Following hybridization, the bound target was washed under stringent conditions to remove non-specific background and to minimize the background noise caused by random ligation events. Each polymorphic nucleotide was queried via a multi-color ligation event conducted on the array surface. After ligation, the arrays were stained and imaged on a GeneTitan MC Instrument (Affymetrix, Santa Clara, CA, USA). The images were analyzed using the Genotyping Console™ Software (Affymetrix, Santa Clara, CA, USA). The genotype data were produced using the K-CHIP available through the K-CHIP consortium. The K-CHIP was designed by the Center for Genome Science at the Korea National Institute of Health (4845–301, 3000–3031).

Samples that revealed the following inclusion thresholds were excluded: sex inconsistency, markers with a high missing rate (>5%), individuals with a high missing rate (>10%), minor allele frequency < 0.01, and a significant deviation from Hardy-Weinberg equilibrium (HWE) (*P* < 0.001). In addition, SNPs that were related to each other in linkage disequilibrium were excluded. The remaining 394,222 SNPs and 2146 samples were used in the subsequent association analyses.

### Statistical analysis

Descriptive statistical analyses were performed using SPSS version 23.0 (IBM, Chicago, IL, USA). The skewed variables were transformed to logarithmic form, and a two-tailed *P*-value <0.05 was considered statistically significant. An independent *t*-test was performed on the continuous variables to compare the parameters between the control group and patients with hypertension. HWE was assessed using PLINK version 1.9 (https://www.cog-genomics.org/plink2). The association between SNPs and BP were evaluated with a linear regression analysis. The frequency was tested using a chi-square test. The association of hypertension with a genotype was calculated using the odds ratio (OR) [95% confidence interval (CI)] of a logistic regression model with an adjustment for confounding factors. A one-way ANOVA followed by a Bonferroni post hoc test was performed to compare the differences among the *EPHA6* rs4857055 C > T genotype groups in the control group and patients with hypertension.

## Results

The clinical and biochemical characteristics of normotensive controls (*n* = 1605) and hypertensive patients (*n* = 541) are shown in Table 1. Case subjects were significantly older and heavier and had significantly higher systolic and diastolic BP, triglyceride, glucose, insulin, and HOMA-IR and lower HDL-cholesterol than controls (Table 1). After adjusting for age, sex, and BMI, hypertensive patients showed significantly higher systolic and diastolic BP, triglyceride, total cholesterol, apo B, and glucose and lower apo A-I than normotensive controls (Table 1).

**Table 1:** Clinical and biochemical characteristics in normotensive controls and hypertension patients

	Normotensive controls	Hypertension group	Adjusted
	(*n* = 1605)	(*n* = 541)	*P*-value
Age (year)	48.0 ± 10.9	54.3 ± 11.6**	–
BMI (kg/m^2^)	23.7 ± 2.91	25.4 ± 3.20**	–
Weight (kg)	63.0 ± 10.2	68.1 ± 11.9**	0.092
Waist (cm)	83.5 ± 7.79	87.8 ± 8.70**	0.690
Waist hip ratio	0.88 ± 0.06	0.90 ± 0.06**	0.130
Systolic BP (mmHg)	116.4 ± 11.5	138.5 ± 15.3**	< 0.001
Diastolic BP (mmHg)	72.7 ± 8.72	87.4 ± 10.7**	< 0.001
Triglyceride (mg/dL)^*a*^	119.7 ± 73.9	148.4 ± 87.5**	0.005
Total cholesterol (mg/dL)^*a*^	198.0 ± 36.0	198.3 ± 36.0	0.017
HDL cholesterol (mg/dL)^*a*^	53.9 ± 13.5	50.4 ± 12.8**	0.450
LDL cholesterol (mg/dL)^*a*^	121.0 ± 32.7	119.1 ± 32.7	0.050
Apolipoprotein A-I (mg/dL)^*a*^	156.1 ± 28.2	155.4 ± 27.9	0.023
Apolipoprotein B (mg/dL)^*a*^	104.4 ± 29.3	104.6 ± 27.7	0.049
Glucose (mg/dL)^*a*^	95.7 ± 20.7	103.9 ± 25.8**	0.002
Insulin (μIU/dL)^*a*^	9.08 ± 4.66	9.83 ± 5.76*	0.609
HOMA-IR^*a*^	2.15 ± 1.29	2.55 ± 2.03**	0.143
LDL particle size (nm)^*a*^	23.9 ± 1.07	23.9 ± 0.80	0.327

Mean  ±  SD. ^*a*^tested by logarithmic transformation. *P < 0.05, **P < 0.001 derived from an independent *t*-test between two groups. Adjusted *P*-value derived after adjusting for age, sex, and BMI

The 394,222 SNPs and 2146 samples were used in subsequent analyses. The associations between genotypes and BPs were evaluated with linear regression analysis after adjusting for age and sex. From the twenty-five most strongly associated SNPs for hypertension, *EPHA6* rs4850755 was the most highly associated SNP with both systolic BP (*P* = 2.63E-08) and diastolic BP (*P* = 3.67E-05); therefore, we performed an association analysis on rs4857055 in *EPHA6* (Additional file 1: Table S1).

### Distributions of the *EPHA6* rs4850755 C > T polymorphism

The genotype distributions of *EPHA6* rs4857055 C > T polymorphism were in HWE. Among 1605 control subjects, 446 individuals (27.8%) had the CC genotype, 829 (51.7%) had the CT genotype, and 330 (20.6%) had the TT genotype. The allele frequency of the T allele was 0.464 in normotensive controls, while among 541 case subjects, 135 individuals (25.0%) had the CC genotype, 261 (48.2%) had the CT genotype, and 145 (26.8%) had the TT genotype. The allele frequency of the T allele was 0.509 in hypertensive cases. The relative *EPHA6* rs4857055 C > T genotype (*P =* 0.010) and allele frequencies (*P =* 0.010) in hypertension patients differed significantly from those in the controls.

The presence of the TT genotype of the *EPHA6* rs4857055 C > T SNP was associated with a higher risk of hypertension [OR 1.415 (95% CI 1.129–1.733), *P =* 0.003] (Table 2). The significance of association remained after adjusting for age, sex, BMI, smoking, and drinking [OR 1.533 (95% CI 1.200–1.959), *P =* 0.001].

**Table 2:** Unadjusted and adjusted odds ratios for all of the patients with hypertension according to the *EPHA6* rs4857055 genotypes

	Hypertension (*n* = 541)	*P*-value
*EPHA6* rs4857055	OR (95% CI)	
Model 1		
C^*a*^ compared with T	1.199 (1.045, 1.377)	0.010
CC + CT^*a*^ compared with TT	1.415 (1.129, 1.773)	0.003
CC^*a*^ compared with CT + TT	1.157 (0.926, 1.447)	0.200
Model 2		
C^*a*^ compared with T	1.220 (1.052, 1.415)	0.009
CC + CT^*a*^ compared with TT	1.533 (1.200, 1.959)	0.001
CC^*a*^ compared with CT + TT	1.134 (0.892, 1.442)	0.305

^*a*^Reference. *CI* Confidence interval. Model 1: unadjusted; Model 2: adjusted for age, sex, BMI, smoking status, and drinking status

### BP associated with the *EPHA6* rs4857055 C > T genotype

There were no significant genotype-related differences among control subjects or hypertensive patients treated without or with antihypertensive therapy according to the *EPHA6* rs4857055 C > T genotype with respect to age, sex, BMI, smoking, and drinking (data not shown). In the control group, significant associations were observed between systolic BP and the *EPHA6* rs4857055 C > T polymorphism (*P* < 0.001) and between diastolic BP and the rs4857055 C > T (*P* = 0.016). In the control group, subjects with rs4857055 TT or the CT genotype showed significantly higher systolic and diastolic BP than CC subjects (all *P* < 0.05). In the hypertension group, a significant association was observed between systolic BP and the *EPHA6* rs4857055 C > T polymorphism (*P* = 0.022). In addition, subjects with the rs4857055 TT genotype showed significantly higher systolic BP than CC subjects (*P* = 0.023).

### Lipid profiles, apolipoproteins, and LDL particle size according to the *EPHA6* rs4857055 C > T genotype

In the hypertension group, trends toward associations were observed between serum triglyceride and the *EPHA6* rs4857055 C > T polymorphism (*P* = 0.069), between serum apo B and the rs4857055 C > T polymorphism (*P* = 0.015), and between LDL particle size and the rs4857055 C > T polymorphism (*P* < 0.001). In the hypertension group, subjects with the rs4857055 TT genotype showed significantly higher apo B levels and smaller LDL particle sizes than those with the TC or CC polymorphisms (all *P* < 0.05) (Table 3).

**Table 3:** Clinical and biochemical characteristics in normotensive controls and subgroups of hypertension patients according to *EPHA6* genotype

*EPHA6*	Normotensive controls (*n* = 1605)			Hypertension group (*n* = 541)		
	CC (*n* = 446)	CT allele (*n* = 829)	TT (*n* = 330)	CC (*n* = 135)	CT allele (*n* = 261)	TT (*n* = 145)
Age (year)	47.9 ± 10.9	48.4 ± 10.8	47.4 ± 11.3	54.6 ± 11.1	54.8 ± 11.8	53.1 ± 11.8
Weight (kg)	63.4 ± 9.98	62.7 ± 10.3	63.4 ± 10.6	66.8 ± 11.3	68.7 ± 12.2	68.1 ± 11.7
BMI (kg/m^2^)	23.8 ± 2.89	23.7 ± 2.94	23.8 ± 2.87	25.0 ± 3.21	25.5 ± 3.23	25.5 ± 3.14
Waist (cm)	83.6 ± 7.75	83.4 ± 7.84	83.6 ± 7.71	87.0 ± 8.76	88.1 ± 8.79	88.1 ± 8.48
Waist hip ratio	0.88 ± 0.06	0.88 ± 0.06	0.89 ± 0.06	0.90 ± 0.06	0.90 ± 0.05	0.91 ± 0.07
Systolic BP (mmHg)	114.5 ± 11.5^*b*^	117.0 ± 11.5^*a*^	117.4 ± 11.2^*a*^	136.4 ± 15.9^*b*^	138.0 ± 14.6^*a,b*^	141.3 ± 15.9^*a*^
Diastolic BP (mmHg)	71.7 ± 8.67^*b*^	73.0 ± 8.63^*a*^	73.1 ± 8.95^*a,b*^	86.5 ± 11.0	87.2 ± 10.6	88.7 ± 10.3
Triglyceride (mg/dL)^*∮*^	115.4 ± 64.3	122.3 ± 77.8	119.1 ± 75.9	144.5 ± 79.5^*a,b*^	141.2 ± 78.7^*b*^	165.0 ± 106.2^*a*^
Total cholesterol (mg/dL)^*∮*^	200.0 ± 37.3	197.2 ± 36.2	197.4 ± 33.8	196.0 ± 33.7	198.5 ± 38.5	200.0 ± 33.2
HDL cholesterol (mg/dL)^*∮*^	54.2 ± 12.8	53.7 ± 13.9	54.0 ± 13.6	51.0 ± 12.8	50.8 ± 13.4	49.1 ± 11.7
LDL cholesterol (mg/dL)^*∮*^	122.9 ± 33.3	120.1 ± 33.1	120.7 ± 30.6	116.2 ± 29.2	120.1 ± 35.3	120.1 ± 31.0
Apolipoprotein A-I (mg/dL)^*∮*^	157.3 ± 26.3	155.9 ± 29.6	155.0 ± 27.1	154.1 ± 25.6	155.8 ± 30.2	156.1 ± 25.6
Apolipoprotein B (mg/dL)^*∮*^	107.6 ± 29.3	103.3 ± 29.4	102.9 ± 28.6	100.8 ± 27.2^*b*^	103.2 ± 28.6^*b*^	110.6 ± 25.9^*a*^
Glucose (mg/dL)^*∮*^	95.0 ± 20.1	95.7 ± 19.7	96.4 ± 23.7	104.4 ± 23.9	104.0 ± 27.6	103.5 ± 24.5
Insulin (μIU/dL)^*∮*^	9.32 ± 4.91	8.97 ± 4.64	9.04 ± 4.32	8.99 ± 4.33	10.1 ± 6.39	10.2 ± 5.67
HOMA-IR^*∮*^	2.18 ± 1.24	2.13 ± 1.35	2.15 ± 1.17	2.30 ± 1.32	2.63 ± 2.42	2.63 ± 1.77
LDL particle size (nm)^*∮*^	24.0 ± 0.81	23.9 ± 1.27	24.0 ± 0.77	24.0 ± 0.94^*b*^	24.0 ± 0.66^*b*^	23.5 ± 0.85^*a*^

Mean  ±  SD. ^*∮*^tested by logarithmic transformation. *P*-values derived from a One-way ANOVA. All alphabetical *P* < 0.05 derived from Bonferroni post hoc tests; no significant differences are marked with the same alphabet and significant differences are marked with a different alphabet

## Discussion

The major finding of this study is that the frequency of the *EPHA6* rs4857055 TT genotype was significantly higher in hypertensive patients than in controls, suggesting an association between the *EPHA6* rs4857055 C > T SNP and hypertension. This observation is consistent with the GWAS data for hypertension, which showed an association with *EPHA6* in a recent study of genome-wide gene-sodium interaction analyses on BP [18].

Ephrins, which are cell surface molecules, are ligands of Eph receptors and are classified as A and B subfamilies. Ephrin-As and ephrin-Bs attach to cell surfaces in different ways [20–22]; in general, Eph receptor A members bind preferentially with ephrin-As and Eph receptor Bs with ephrin-Bs. The Eph/ephrin system regulates blood vessel remodeling and stabilization by regulating endothelial cells. EphA2 regulates angiogenesis and vascular permeability mainly in concert with ephrin-A1 through interactions with vascular endothelial growth factor [23, 24]. These findings support the idea that EphA receptors and ephrin-As are involved in blood vessel regulation and suggest that the EphA/ephrin-A system represents a target for the inhibition of angiogenesis via reductions in hypoxia and the vascular changes caused by inflammatory cytokines [11, 12, 25]. Increasing evidence demonstrates an association between Eph/ephrin and neovascularization [26]. A recent microarray analysis on the gene expression profiles of Eph receptors demonstrates that EphA6 mRNA levels are higher in adult human peripheral blood monocytes [9]. These results indicate that a wide variety of ephrins and Eph receptors might affect monocyte chemotaxis, contributing to the development of atherosclerosis. Furthermore, previous studies have identified associations of *EPHA6* with obesity-related traits [27], and the control of glucose homeostasis has emerged as a role of the EphA/ephrin-A system [21].

Hypertensive subjects with the *EPHA6* rs4857055 TT genotype showed higher systolic BP than those with the CC genotype in this study. Hypertension is known to contribute to atherosclerosis and endothelial cell dysfunction, with associated risk factors that influence LDL size [28]. Small LDL particle size and high serum triglyceride or triglyceride-rich lipoproteins and apo B were reported to be found in nondiabetic subjects with essential hypertension [29, 30]. In the hypertension group of this study, subjects with the *EPHA6* rs4857055 TT genotype showed a higher tendency of serum triglyceride and significant increases in apo B than those with the CC or CT genotype.

Endothelial cell dysfunction has been suggested as the initiating process in the development of cardiovascular disease and is considered to be closely related to the pathophysiology of hypertension. Accumulating evidence suggests that hepatocyte growth factor (HGF) plays an important role in endothelial cell dysfunction. The association between HGF and hypertension severity has been established in several human studies [31, 32]. Linked with those previous studies, a Japanese study revealed the association between *HGF* polymorphisms and BP or atherosclerosis and suggested that the *HGF* located at chromosome 7q11.2-q21 is a candidate gene for atherosclerosis [33]. In addition, the interleukin-6 gene plays a role in BP regulation and the progression of atherosclerosis in Japanese individuals [34] by stimulating the proliferation of VSMCs [35], indicating that this cytokine may play an important role in the development of arteriosclerosis.

Most reported functions of Ephs occur in the central nervous system, and some are expressed in endothelial cells [17]. Accumulating studies have demonstrated that VSMCs are the major targets through which Ephs/ephrins exhibit their effect on BP modulation [17, 36–39]. EphB6 and EphB4 regulate VSMC contractility and modulate BP [17], and EphB4 deletion results in hypotension in an animal model [37]. EphA2 and EphA4 are also expressed on VSMCs, with possible effects on endothelial cells and other surrounding cells [40, 41]. EphA6 and EphA7, expressed on vascular endothelium, are also involved in angiogenesis [42, 43]. However, to date, there are no studies on the exact function of EphA6 in VSMC contractility and BP regulation. Based on several studies that have shown that Ephs, particularly Eph A members, are key modulators of BP, we could presume that EphA6 is another important protein for hypertension development. Gordillo-Moscoso et al. [44] demonstrated the positive association between serum levels of triglycerides and vascular inflammation, measured as cyclooxygenase-2, which is highly expressed in VSMCs [45]. Wang et al. [38] revealed that ephrin-B3 knockout in VSMCs leads to attenuated myosin light chain kinase phosphorylation in which enhances Ca^2+^ sensitivity of VSMCs. Recently, the ephrin-B3 gene (*EFNB3*) has been suggested to be a hypertension risk gene in certain individuals [36]. Through previous studies, we can conclude that VSMCs are a target tissue for EphA6 function in BP regulation. In the present study, the *EPHA6* rs4857055 TT genotype showed markedly higher triglyceride levels, a phenomenon (hypertriglyceridemia) that could result in strong VSMC contraction, leading to increased BP.

When interpreting the present findings, it should be noted that our results share the limitations of cross-sectional observational studies, by which we only evaluated associations rather than prospective predictions. Additionally, we specifically focused on a representative group of Korean subjects; therefore, our results cannot be generalized to other ethnic, age, or geographical groups. Further studies are needed to provide a better understanding of the physiological relevance and exact mechanisms of this molecule and its role in BP regulation, which could represent a novel personalized therapeutic approach to BP management. Despite these limitation, our results show an intriguing association between the *EPHA6* rs4857055 TT genotype and increased risk of hypertension.

## Conclusion

The results of this study suggest that the *EPHA6* rs4857055 C > T SNP could be a novel candidate gene for hypertension; moreover, the *EPHA6* rs4857055 TT genotype could be associated with hypertriglyceridemia and small LDL particle size in hypertension.

## Additional file

Additional file 1: Table S1. Top twenty-five SNPs associated with systolic and diastolic BP. (DOCX 17 kb)

## Abbreviations

BMI: Body mass index
BP: Blood pressure
Eph: Erythropoietin-producing human hepatocellular
EPHA6: Eph receptor A6 gene
Ephrin: Eph receptor-interacting protein
GWAS: Genome-wide association studies
HDL: High-density lipoprotein
HOMA: Homeostasis model assessment
HWE: Hardy-Weinberg equilibrium
IR: Insulin resistance
K-CHIP: Korean Chip
LDL: Low-density lipoprotein
SNP: Single-nucleotide polymorphism
VSMC: Vascular smooth muscle cell
