# Supplementary material for: EPHA6 rs4857055 C > T polymorphism associates with hypertension through triglyceride and LDL particle size in the Korean population
Source: Lipids Health Dis. 2017 Dec 6;16:230. doi: 10.1186/s12944-017-0620-5 (PMC5718072; doi:10.1186/s12944-017-0620-5)
Supplement: Additional file 1: Table S1. — Top twenty-five SNPs associated with systolic and diastolic BP. (DOCX 17 kb) [file 12944_2017_620_MOESM1_ESM.docx]

**Table S1. Top twenty-five SNPs associated with systolic and diastolic BP.**

| **No.** | **Chr** | **Associated gene** | **SNP** | ***P*-value** |
| --- | --- | --- | --- | --- |
| **Systolic BP** | | | |  |
| 1 | 19 | *TPRX1* | --- | 3.99E-17 |
| **2** | **3** | ***EPHA6*** | **rs4857055** | **2.63E-08** |
| 3 | 1 | *SRRM1, CLIC4* | rs117559502 | 9.65E-07 |
| 4 | 21 | *LINC00478, C21orf37* | rs116861740 | 1.20E-06 |
| 5 | 12 | *AMN1* | rs142983199 | 4.27E-06 |
| 6 | 18 | *SERPINB7, SERPINB2* | rs62099117 | 5.52E-06 |
| 7 | 5 | *PAM, LINC00491* | rs13175330 | 6.77E-06 |
| 8 | 10 | *ARHGAP22* | rs151307475 | 1.04E-05 |
| 9 | 18 | *LOC100505817, FBXO15* | rs4280345 | 1.09E-05 |
| 10 | 12 | *KRAS, LMNTD1* | rs12828533 | 1.15E-05 |
| 11 | 21 | *FLJ42200, LOC339622, LOC101927869* | rs74353152 | 1.33E-05 |
| 12 | 8 | *SNTB1* | rs151310375 | 1.60E-05 |
| 13 | 16 | *RBFOX1* | rs117332112 | 2.12E-05 |
| 14 | 3 | *ERC2* | rs9848499 | 2.15E-05 |
| 15 | 11 | *NAV2* | rs1559665 | 2.18E-05 |
| 16 | 9 | *FOXD4, LOC101929127, LINC01388* | rs7864555 | 2.23E-05 |
| 17 | 8 | *HAS2, SNTB1* | rs75263934 | 2.59E-05 |
| 18 | 8 | *ENPP2, TAF2* | rs77079853 | 2.66E-05 |
| 19 | 13 | *TNFSF13B, MYO16* | rs146435589 | 2.77E-05 |
| 20 | 10 | *MGMT, LINC01163, LOC105378555* | rs4751007 | 2.88E-05 |
| 21 | 3 | *SLC6A20* | rs7633172 | 3.24E-05 |
| 22 | 3 | *ACTL6A, GNB4, LOC107986157* | rs4855081 | 3.48E-05 |
| 23 | 6 | *ASCC3* | rs6904959 | 3.57E-05 |
| 24 | 9 | *NFIB* | rs17213362 | 3.59E-05 |
| 25 | 16 | *FTO* | rs116930710 | 3.61E-05 |
| **Diastolic BP** | | | |  |
| 1 | 19 | *TPRX1* | --- | 1.20E-14 |
| 2 | 5 | *PAM* | rs13175330 | 6.04E-07 |
| 3 | 1 | *RGS7* | rs1915872 | 1.11E-06 |
| 4 | 22 | *TOP3B, VPREB1* | rs6001482 | 1.66E-06 |
| 5 | 7 | *NOBOX* | rs12539814 | 3.83E-06 |
| 6 | 19 | *SLC5A5* | rs149026664 | 7.07E-06 |
| 7 | 12 | *LINC00936, LINC00615* | rs17192198 | 8.36E-06 |
| 8 | 11 | *NAV2* | rs1559665 | 1.21E-05 |
| 9 | 22 | *PVALB* | rs9607382 | 1.45E-05 |
| 10 | 4 | *LOC101927282, C4orf33* | rs117986095 | 1.82E-05 |
| 11 | 11 | *MMP13, DCUN1D5* | rs17099821 | 1.92E-05 |
| 12 | 3 | *SEMA5B* | rs9839593 | 2.02E-05 |
| 13 | 9 | *ADAMTS13* | rs186954413 | 2.10E-05 |
| 14 | 12 | *SRRM4, HSPB8* | rs73412041 | 2.31E-05 |
| 15 | 8 | *LAPTM4B* | rs35733152 | 2.34E-05 |
| 16 | 11 | *OR5M11, OR5M8* | rs117840803 | 2.73E-05 |
| 17 | 14 | *AREL1, LTBP2* | rs2098499 | 2.97E-05 |
| 18 | 20 | *TOX2* | rs11700304 | 3.01E-05 |
| 19 | 3 | *SLC6A20* | rs758386 | 3.01E-05 |
| 20 | 3 | *LOC101927347, LOC105376938* | rs151242956 | 3.09E-05 |
| 21 | 3 | *LOC101927494, VGLL3, RNU6-69P* | rs189324335 | 3.35E-05 |
| 22 | 11 | *JRKL-AS1* | rs73524843 | 3.57E-05 |
| **23** | **3** | ***EPHA6*** | **rs4857055** | **3.67E-05** |
| 24 | 5 | *TENM2* | rs11744503 | 3.83E-05 |
| 25 | 6 | *PRR18* | rs9356436 | 4.47E-05 |
